# “It’s What I Have, It’s Not Who I Am”: A Qualitative Study of Social Support in Education/Employment Settings and Transition Readiness of Young Adults with End-Stage Renal Disease

**DOI:** 10.3390/ijerph18126596

**Published:** 2021-06-19

**Authors:** Sophie Rupp, Cynthia Fair, Hannah Korycinski, Maria Ferris

**Affiliations:** 1 Department of Public Health Studies, Elon University, Elon, NC 27244, USA; sophielrupp@gmail.com (S.R.); hkorycinski@elon.edu (H.K.); 2 UNC Self-Management and Transitions (STARx) Program, UNC-Chapel Hill, Chapel Hill, NC 27599, USA; maria_ferris@med.unc.edu; 3 The UNC Pediatric Diagnostic and Complex Care Program, UNC-Chapel Hill, Chapel Hill, NC 27599, USA

**Keywords:** end-stage renal disease, ESRD, social support, transition, self-management, young adults, education, employment

## Abstract

This study investigated the role of social support in self-management within education/employment settings for young adults (YA) with end-stage renal disease (ESRD) as well as barriers and facilitators to social support formation. Nineteen YA with ESRD (mean age 24 years, 10 males, 9 African American) recruited from a pediatric nephrology clinic in the Southeast United States completed in-person semi-structured interviews. The grounded theory was used to analyze transcribed interviews to identify emergent themes. Absences hindered participants’ school/work attendance and performance. Social support was necessary for illness management and success in academic/vocational settings. Facilitators to establishing support included self-awareness and view of disclosure as a way to access accommodations. Barriers included fear of judgment, job loss, and the belief that the condition was too personal to disclose. Educators and employers must acknowledge the needs of YA with ESRD to promote development and educational/vocational success. Fear of disclosure and poor disease self-management interferes with accessing social support. Communication skills and autonomy in patients’ medical and personal lives can promote success in education and employment settings.

## 1. Introduction

In the United States, about 144,000 young adults (YA) between ages 18 and 29 currently live with end-stage renal disease (ESRD; United States Renal Data System, 2014) [1]. Patients with ESRD, a chronic condition that affect cognition and quality of life, require renal replacement therapy (dialysis or transplant). ESRD is a cumbersome condition to manage as it has many co-morbidities that require multiple medications and a special diet. Unlike adults, most pediatric ESRD patients receive a kidney transplant, which then requires continuous medication and monitoring [2]. Studies demonstrate that this population has difficulty living independently [3], less likelihood of completing key developmental milestones [4,5], higher levels of unemployment [6], and lower levels of education [7,8]. To understand these issues, it is necessary to investigate a patient’s autonomy, self-management abilities, and the support systems they create to promote independence.

For YA who receive treatment in a pediatric setting, this process is viewed through the lens of healthcare transition (HCT), defined as ‘‘the movement of adolescents and young adults with chronic physical and medical conditions from child-centered to adult-oriented healthcare systems’’ [9] (p. 570). Inadequate HCT preparation is often associated with poor clinical and psychological outcomes, as patients must handle challenges related to their condition and those typical of this developmental period [10,11]. While HCT can encourage patients to take an active role in their medical care, the process often fails to emphasize psychosocial development, emotional health, and educational or vocational aspirations [12,13].

Within the existing HCT literature, there are limited evidence-based transition strategies [14,15]. Furthermore, since the pediatric ESRD population is relatively small, nephrologists in adult care may be less familiar with general HCT strategies for YA with ESRD and those related to their academic or professional success [15,16]. Some studies identify successful transition outcomes for transplant recipients, such as knowing the cause of kidney failure, awareness of the implications of renal transplantation, managing appointments and medications, and accessing health insurance [17,18]. These behaviors constitute transition readiness, and given their influence on an individual’s independence and health, practitioners have begun constructing models to promote their development.

The majority of HCT models implemented in clinical settings emphasize characteristics of patients, providers, and medical systems and the best coordination strategies among them [19,20]. A model proposed by The International and Interdisciplinary Healthcare Transition Research Consortium (HCTRC) [10] offers a theoretically driven and comprehensive approach to HCT in four influential domains: Individual, Family/Social Support, Health Care System, and Environment. While the model contains categorical divisions, it also allows for overlap between them, as it accounts for the complexity of variables that facilitate or hinder successful HCT. Among these categories, there are a limited number of studies regarding experiences of YA with chronic illness within the Environment Domain. This realm includes secondary and postsecondary educational opportunities, employment, and community supports. Moreover, educational and professional attainment depend upon a patient’s ability to manage their health needs, access mechanisms of support, and build connections that allow them to obtain health-related accommodations. This domain was explored in Javalkar et al.’s study [21], in which researchers found that characteristics of chronically ill YA’s environments, such as median income, language, and sex composition of their surrounding community, can impact self-management and transition readiness. While this study examines how specific ecological factors may affect patients, it does not investigate how self-management skills affect the ways YA interact with their environments, particularly those related to education and employment.

Additionally, it is important that further qualitative research not only examines the functionality of this population in school or at work but also incorporates and links these findings to HCT. Doing so requires going beyond measures of education and employment to understand these outcomes phenomenologically. Generally, scholars in the field of pediatric/adolescent medicine and HCT suggest that improving the academic and professional success of renal patients can occur by investigating two major areas: self-management and social support [4,6,10,22].

HCT readiness can be achieved through the development of self-management skills or the ability to take full responsibility for maintaining physical and psychological well-being [14]. Few studies examine YA renal patients’ self-management behaviors in the context of academic and professional settings. One study by Murray et al. employed semi-structured interviews to find illness-related barriers in school and work settings confronted by YA with ESRD using dialysis [22]. These included difficulties maintaining a consistent work or school schedule that accommodated appointment times. Moreover, the unpredictable effects of dialysis, such as fatigue and low mood, presented challenges to work or school-related activities. This claim is supported by Van der Mei et al.’s interview-based study, which also identifies physical symptoms as a barrier to renal patient functionality in the workplace [23]. Additional research must not only explore these barriers but also seek to understand how YA confront them. Within HCT, scholars emphasize the need for further research on the relationships this population forms with individuals or systems that allow them to access accommodations that promote self-management [4,5,24,25,26].

One consistently identified facilitator of self-management is social support or a network in which adolescents can give and receive affection and aid [27,28]. This theory is supported by the literature emphasizing relationships between authoritative figures and disabled individuals as key determinants in productivity, self-esteem, and access to health accommodations [29]. Unfortunately, it was well established that children with ESRD may have difficulty creating social support networks due to social isolation and heavy dependence on family [4,29,30]. Although these problems can persist into adulthood, little research examines patients’ competency to create social networks and articulate their needs for illness-based adjustments to authoritative figures as they start school programs or jobs. Disclosure about ESRD is difficult for young adults because they find it challenging to bring it up in conversations in their relationships and worry about their partners’ reactions [31]. A study conducted by Murray et al. reveals patients’ tendency to conceal their ESRD from employers for fear of job loss and perceived lack of understanding [32]. Many young adults with ESRD have difficulties attaining higher education and employment because they perceived their disease as getting in the way [7,32]. Maintaining employment is exceedingly difficult as young adults with ESRD progress and manage their disease [33]. These experiences must be studied further to understand how patients manage their illness without communicating their needs to authority figures. Furthermore, it is important to understand why, if at all, patients do choose to communicate their needs to authority figures and the dynamics of such interactions.

### Current Study

Although social support was identified as a factor that could potentially facilitate self-management in these settings, few studies have examined ways YA with ESRD establish and utilize these networks. Furthermore, limited research has explored the context of transition outside of the medical sphere. This project aims to explore these realms by investigating the relationship between social support in education/employment settings and transition readiness for YA with ESRD.

## 2. Methods

Participants: This qualitative cross-sectional study included a purposive sample comprised of nineteen YA (mean age, range 18–29 years) who had experienced renal failure prior to the age of 21 and received care in the pediatric clinic participated in face-to-face semi-structured interviews. The sample was obtained from a list of pediatric nephrology patients who received care at a large teaching hospital in the Southeast United States. Patients were eligible for the study if they could communicate in English, did not have severe cognitive limitations (as deemed by their providers), and did not suffer from mental health conditions that would limit their ability to complete the interview. Of the 23 contacted, 19 (82.6%) agreed to participate. Four declined because they were either too busy or did not feel comfortable discussing their disease.

Interviews were conducted by a female researcher (SR) trained in qualitative data collection. In addition to formal training, SR shadowed medical providers from the hospital clinic and attended multiple meetings where research related to ESRD was discussed and shared.

Procedure: Institutional Review Boards at Elon University and the University of North Carolina approved the project. After phone contact was made and informed consent was gathered, face-to-face semi-structured interviews lasting 30–45 min were conducted at public locations convenient for the participant. Participants received a $40 gift card as a token of appreciation. The interviews were recorded and transcribed.

Interview: The interview included basic demographic information and self-reported history of ESRD. Next, the interview explored the effects of ESRD on education/employment, self-management and accommodations in school/work settings, disclosure, adjustments, and advice for others. The questions in the interview were guided by the model proposed by the HCTRC [10]. We specifically developed open-ended questions that asked participants to share their lived experiences of navigating in education and employment settings while living with ESRD (See Supplementary for interview guide).

Analysis: Quantitative demographic information was analyzed using Microsoft Excel (Microsoft Corpration, Redmond, Washington). Univariate analyses were conducted. A grounded theory method was used to analyze qualitative data. This method employs an inductive strategy in which comparisons among experiences allow researchers to identify emergent themes and generate theory rather than confirm predetermined hypotheses [34]. This technique enhances the accuracy of analyses, as theories created directly from data can more closely capture reality than comparisons between data and speculative conjectures. In addition, this strategy can “offer insight, enhance understanding, and provide a meaningful guide to action” (p. 12). The grounded theory was appropriate for this project given the multi-faceted nature of ESRD, as the disease is associated with various causes, symptoms, treatment modalities, and onset-ages that are experienced by some patients and not others. Thus, the framework was effective in illuminating commonalities among diverse illness narratives.

Upon completion of the transcription, the interviews were compared to the original audio recordings to ensure accuracy. Transcripts were entered into Dedoose, a qualitative data analysis software program (Dedoose V6.1.18) [35]. Using Dedoose, authors read through transcripts in their entirety and completed an open-coding process, allowing for the construction of codes that categorized these experiences [34]. Throughout this process, the authors (CF and SR) often met to discuss findings until they came to a consensus regarding codes and categorical designations for the data.

Next, a member check was employed. A member check is a “procedure that can be used to determine if data and findings reflect the respondent’s realities” and can confirm the reliability and validity of qualitative data [36] (p. 523). Since member checks work to reduce error in the depiction of multiple, constructed realities, suggestions for revisions can be incorporated to reflect participant’s experiences more accurately [37]. To do so, the authors contacted previous participants to review preliminary themes, interpretations, and direct quotations. Two participants responded to the invitation. Finally, the results were also shared with the team of medical providers from the participants’ clinic who are intimately familiar with the challenges experienced by YA with ESRD. Both research participants and team members responded that the analyses accurately represented the wide variety of experiences of youth living with ESRD. No changes were recommended.

## 3. Results

The sample characteristics of the YA are summarized in Table 1. The mean age of participants was 24 years (range 19–28). Nine participants identified as African American, and the majority were male (n = 10). Many (n = 8) lived with their parents, and 12 had completed some college education or vocational school. Six participants were employed: Five were employed in a service industry (e.g., cashier), and one as an emergency operator.

Most participants (n = 15) reported using dialysis, and the average age of dialysis onset was 20.1 years. Ten reported that they had received hemodialysis. Five had ever received a transplant. Of these participants, two had experienced transplant loss. See Table 2 for additional disease-related characteristics details.

The qualitative analyses revealed that YA wanted to attend school or work yet encountered barriers to doing so. The establishment of social support in these settings helped participants overcome barriers and improved education and employment experiences. The formation of support networks was rooted in an individual’s personal relationship with ESRD and followed stages of identity construction and self-acceptance. After grappling with this process, most YA gained a greater awareness of their condition, limitations, and readiness to re-enter the workforce or an academic program. Many identified the formation of social support through disclosure of their illness as having an impact on their pursuits once they assessed their limits. Participants were able to request accommodations from relevant authority figures by using effective communication styles. Despite an overall emphasis on the importance of social support, many still faced barriers to creating networks.

Willingness to attend school/work

Almost all participants displayed a willingness to pursue education and employment opportunities as they sought to gain a sense of productivity. A 28-year-old female stated, “I’m just tired of sitting home […] I just want to forward my education.” Another young woman was motivated to seek employment when she began living with her partner, mirroring the sentiments of many YA who wanted to support their families:

*He pays for most of the stuff, and it takes away your independence when you’re relying on someone else. We rent a house together. We have cars and just the basic necessities of a house, the energy, and all that stuff. When he pays all that, it makes me feel like I’m just sitting there. I’m not helping. I’m not contributing*.

Barriers to school/work attendance

When discussing their current employment or level of education, many YA felt that absences associated with their condition acted as a barrier to their productivity and pursuit of academic or professional endeavors. Analyses revealed two kinds of absences that prevented school/work attendance: short absences and long-term absences followed by the process of restarting a program or job.

*Short Absences:* The majority of illness-related barriers faced by YA were frequently occurring physical issues, including fatigue following dialysis and side effects of medications. Additionally, routine appointments and dialysis schedules made it difficult for participants to maintain consistent employment hours and a level of functionality at which they could work and attend school regularly. The short-term impact of dialysis is revealed in one 26-year-old female’s description of the challenges the treatment’s physical effects presented to her education:

*It was very hard, first with your dialysis schedule, and then you just have your days. Some days you just don’t feel well. There are days when you feel like crap, but you just have stuff to do. I took on the challenge and my professor knew, but I guess I can’t make an excuse every day if I don’t feel well.*

*Long-term absences and restarting:* Several were forced to drop out of school or take extended absences due to long-term physical problems such as low renal function and transplant rejection. At times, dialysis schedules also caused YA to take breaks from their education. A 24-year-old male recounted, “I was thinking about going back to school, and I’m not sure because going to dialysis three times a week, it drains you.” Even when their health improved, many were hesitant to return to an academic setting for fear of losing motivation or being unprepared.

*It’s hard to be out of school for a little bit and then try to go back sometimes. If you stay out too long, when you come back you won’t be motivated (21-year-old female).*

*Sometimes when you’re out of school for seven, eight years, it’s scary to go back. Homework and teachers, college is different. Nobody is standing over your shoulder wondering if you’ll succeed or if you’ll fail. You have fears (25-year-old female).*

This sentiment was also echoed by a 22-year-old male participant who, at the time of the interview, was not enrolled in an academic program. He not only expressed his hesitancy to return to school but also voiced his fears related to the financial implications of restarting and possibly stopping again.

*Try to go back out, try to do it again and then you just get sick and have to start all over again and pay all the money back again. Just the cycle back over again. Something you don’t really want to do.*

Overcoming barriers through social support One crucial aspect of the transition that allowed patients to overcome challenges in school or work settings was the formation of social support. Through interviews, it was evident that the complex process of support formation often involved a series of stages or phases grounded in the individual’s personal relationship with their illness. Before they felt comfortable discussing their disease with others, some YA struggled to understand how their condition influenced their identity. Greater personal development not only helped many YA accept their condition but also gave them better awareness regarding their needs and readiness to attend school or work. Most importantly, an understanding of these boundaries and the need for support improved participants’ confidence in disclosing their disease to relevant authority figures. Furthermore, they described effective communication styles to facilitate the process. Ultimately, by discussing their disease and forming networks of support, YA were able to access accommodations that allowed them to manage their disease in a given environment. It is important to note that the path each individual took to overcoming these barriers is unique. While the process at times progressed in a general trend, these stages of personal development were often fluid, and the order in which they were experienced varied.

*Identity and self-acceptance:* Many grappled with their classification as a renal patient or the fear of being pitied for their condition.

*I didn’t want to tell anybody because I was kind of ashamed. First of all, I was young, and I didn’t want to be like, “My kidney’s failing.” That word came all weird to me at the time. Maybe they would have understood, but I didn’t tell anybody (24-year-old male).*

*People will either feel really sorry for you or kind of just think, “you’re about to die anyway.” People are really mean that way (23-year-old female).*

YA revealed two major strategies for accepting the ways ESRD influenced their identity. First, to combat a sense of shame, several adopted a mindset in which they did not concentrate on being “sick” and refused to be pitied by others or themselves. Almost one-third of participants found that they could remain positive and motivated to pursue their goals with this mindset. While discussing her future plans and ways ESRD might affect them, one 23-year-old female stated:

*I act like I don’t have the kidney disease. It helps. Even though in the back of my mind I know I do, but I try to go through my daily activities and go and plan things. Say “nothing’s going to hold me back,” and more than likely, it won’t. If it does, if a problem presents itself, I’ll find a way around it just like I do with everything.*

Another young man described a similar outlook he maintained that ultimately gave him the motivation to graduate college:

*I didn’t let it hold me down or define me, and I didn’t look for anybody’s pity because of it. […] I had my pity party, and I realized that I could either continue down this path and be depressed, and […] lose everything I had going for me, or buckle up, realize, yep, you’re sick, but you’re not dying, you ain’t dead yet, people have it worse, and there are things you can do to be better, so do it (24-year-old male).*

The second mechanism YA used was to conceptualize their classification as a renal patient as a part of their identity but not as a solely defining characteristic.

*Even with all the dialysis and the transplant thing, it’s what I have, it’s not who I am. My thought process was that if I talk about it, there’s nothing to be worried about, there’s nothing to be embarrassed about (24-year-old male).*

*Awareness and readiness:* Participants described how, through self-awareness, they were more adept at assessing multiple aspects of their health, allowing them to make realistic decisions regarding their readiness to attend school or work. One 26-year-old female recalled how her personal motivation to work drove her to quickly accept a job. Unfortunately, her dialysis regimen and depression left her unable to perform well or maintain her health. Reflecting on the experience, she advised:

*You just have to do it when you’re ready, not when other people are ready, and other people are pushing you. You have to do it when you’re ready because you know your body, and you know what you’re going through mentally and physically. You just have to do it on your time.*

Another participant, feeling pressure to complete her education before her condition worsened, started college and a new job immediately after high school. After dropping out, she realized how important it was to assess her health before starting another program or work experience:

*The most important things to know is, you have to know your body at all times. Especially when you’re in dialysis, you’ve got to keep up with every single thing that they do. […] I thought that I could work through school and figure all this out. It’s impossible, and you’re just going to kill yourself and your levels... Your levels reflect when you’re stressed. Big time. So, just pick one [opportunity] and go for it (21-year-old female).*

When discussing their futures, five YA emphasized the role of self-awareness in planning and researching school or work opportunities that best fit their physical abilities. One young man aspired to work as an EMT, yet realized that he would be unable to do so given the position’s physical requirements. However, he pursued his interest in emergency services by discussing his condition with his providers and others who were familiar with the field. Ultimately, he was able to find a job that was still within his realm of interest and accommodated his limited physical capacity.

*Taking what I’ve learned from having to withdraw from college, as far as not being able to meet the requirements for clinical, knowing my risk for infection in talking to my nephrologist through the dialysis process, and my nurse through dialysis whose husband was a paramedic, it was just very clear that it wasn’t going to be something that was easy to do. I wasn’t willing to do all that. Working 12 h shifts and getting hooked to the machine for nine hours was going to be rough, so I just decided let’s not do the ambulance thing. Let’s get hired in the call center, put our time in, and move on from there (24-year-old male).*

*Understanding the need for social support:* By becoming more cognizant of their issues, many YA understood their responsibility to seek the care and support they needed, as noted below.

*Do what you can do to help yourself. Don’t fall into the pity party trap, and realize that there are resources out there, people that you can talk to. The biggest resource you have is yourself, and if you lose all drive to help yourself, nobody else is going to help you (24-year-old male).*

Specifically, participants emphasized the importance of discussing their illness with employers or educators. On the one hand, disclosure was deemed crucial in preparing for emergency situations. One 26-year-old female explained:

*I told people at work just in case one day something bad were to happen, and they needed to call a doctor, they would know what was going on.*

Declining kidney function also motivated others to initiate conversations about potential self-management behaviors/accommodations:

*When I hit 20% kidney function, that is when they start the kidney transplant process. That is when I had to go talk to my manager basically about, you know, at some point, I could be missing some work (24-year-old male).*

Others felt that disclosure was necessary to explain their preventative and general self-management behaviors. Outside of making adjustments to work environments and schedules, participants stated that disclosure spurred conversations regarding Family and Medical Leave Act benefits and matters of insurance.

*Effective communication with authority figures to establish supports:* YA asserted that barriers to attending school and work could be overcome by establishing supports with authority figures in these settings. For some, doctors or other health care providers communicated with a participant’s employer or educator. The majority preferred direct, personal communication with both individuals and larger systems such as a human resources department. YA also mentioned that communication was easy if an educator or employer made an effort to stay informed of their needs.

*If you’re in a job setting, then you need to go talk to your HR. […] You should be able to talk freely about it. I’m not saying run around your job and tell everybody your problems. Most jobs anywhere you go claim they have an open-door policy. At any given time, you should never be embarrassed to go in there and say, “Hey, I’m having this problem. How can we work this out?” Most of the time, they work with you (25-year-old female).*

*I mean, just try to work with them as much as they work with you. You know. That’s the thing, it’s a two-way street. If I have a disease and I’m not working with the teacher, the teacher has no reason to work with me (24-year-old male).*

*Accommodations requested from authority figures:* Participants indicated that they used the above communication styles to request a range of accommodations necessary at school and work. In academic settings, specific needs included longer time to walk between classes, rescheduling tests, use of alternative ways to obtain assignments during absences, time to take the medication in private areas, and extended due dates for assignments during periods of low energy, fatigue, severe pain, headaches, and other short-term physical problems. While at work, adjustments included asking for tasks that allowed for sitting rather than standing and that did not require heavy lifting, time to drink water and take frequent bathroom breaks, and avoidance of extended time in the sun. In both settings, participants asked for time off to attend appointments and dialysis sessions.

Barriers to establishing social support Despite the importance of disclosure for access to accommodations and resources, YA identified three barriers to accessing social support in education and employment settings: feeling that the disease was too private a matter to disclose, job loss (feared and experienced), and judgment of others.

*Too personal:* Almost half of YA admitted to feeling that the disease was too personal to disclose in school or work settings, and every participant admitted to having this experience at least once during the course of their illness.

*I’m a little bit self-conscious about it, but then I’m not. Like how I got the catheter and stuff, I do all I can to hide it so nobody can see it. I just don’t like talking about it. For one, I’m real private. I don’t like a lot of people knowing about me. If they know about it and they ask me about it, I’ll tell them, but then I’ll tell them, “Don’t tell nobody.” (22-year-old male).*

*To be honest, it’s none of their business because then they’re going to ask a thousand more questions, and I’m not going to feel like answering (28-year-old female).*

Oftentimes, participants did not recognize the need for help from others outside immediate support circles:

*I’m very personal when it comes to certain stuff. I don’t really like to tell professors or employers, or anyone else. My main support knows, and that’s about as far as it goes (21-year-old female).*

*Job loss:* Many participants feared that disclosure would threaten their chances of securing employment, as insurance costs and absences would make them less desirable workers. Four had actually experienced a job loss or were not hired following conversations regarding their condition. These participants noted:

*I got rejected for a job because I came out forward and told them upfront, and they told me no (26-year-old female).*

*I can go and fill out for a job and I’m hesitant to tell them I’m going to have doctors’ appointments. So it’s hard. When I open up and tell them, it’s just a lot, and they’re like, okay, she’s not going to be here, so we’re not going to waste our time on her. (26-year-old female)*

*Judgment:* Some individuals refrained from disclosing their kidney disease for fear of misjudgments regarding their skills or ability to work.

*I feel like they would judge me differently. ‘She can’t do this; she can’t do that. She’s feeble; she can’t lift heavy objects.” I won’t tell them, get the job, tada, everything’s fine (26-year-old female).*

One 22-year-old male emphasized that his ability to perform tasks was at the same level as others.

*People like us are hardworking. Been through pain and through suffering but made it through and do it just as well as anybody else can do it. Might miss days, might have doctor’s appointments, get sick. But that’s not going to stop us from still working as best we can.*

## 4. Discussion

This study’s aim was to investigate the relationship between social support and self-management in employment and education settings for YA with ESRD. Qualitative analysis revealed that although the majority of participants expressed a strong desire to work and attend school, one barrier to doing so was appointment-related absences. In addition, side effects associated with medication and dialysis were identified as deterrents confirming previous research that the impacts of dialysis discourage school and work participation [24,32].

The current study and extant literature document variability among experiences regarding the formation and utility of social support with authority figures. We determined that diversity exists because an individual’s unique and personal interpretation of the disease itself often shaped their attitude toward disclosure. The process of interpreting and accepting one’s illness is complex. Given the unpredictability of ESRD expression, personal conceptions of the disease are often dynamic; the progression of this interpretation is frequently non-linear. Participants in the current study often moved between these stages of identity conceptualization based on the status of their condition, with acute problems, in particular, acting as a catalyst for understanding their limitations and viewing support for self-management purposes. The dynamicity of illness perception in renal patients was also observed by Kierans and Maynooth, who determined that new sensory experiences in the form of pain, transitions between treatment modalities, or changes in interactions with one’s surroundings could shift to alter the illness experience [38]. Our findings delve further into these shifts by identifying certain stages of change, the ways they can reflect a personal view of disease, and how they may influence the formation of social support.

The analyses revealed that patients reconcile their disease status as part of their identity by either choosing not to fixate on this aspect of their person or to learn to define themselves in another way. As participants found ways to accept themselves, they no longer had to focus on rejecting their illness and its inclusion in their lives. Instead, they were better able to assess their capabilities and eventually engage in problem-solving. The most direct result of this process was an understanding of the need for disclosure, as discussing limitations and necessary accommodations with authority figures emerged as a crucial strategy for functioning within a school or work setting. Although other scholars have also determined that general disease knowledge promotes adherence in renal patients [38,39], our study is unique in that it links identity, knowledge, and adherence through the formation of social supports in specific environments. Furthermore, interviews conducted by Kierans and Maynooth revealed that rejection of classification as a “renal patient” could drive nondisclosure [38]. The current study expands upon this idea suggesting that disease acceptance can promote disclosure. Cerrato, Avitable, and Hayman found that adult transplant patients who remained optimistic and concentrated on their abilities rather than their limitations could more easily reject a “sick role” and increase their work performance and social activity [40]. Our findings also support the notion that renal patients who perceive themselves as unconstrained by illness can adopt a more positive view of their identity and challenge the idea that they are too limited to pursue education or employment.

YA explained how disclosure to an authority figure within a school or work environment was a necessary step in obtaining accommodations and ensuring safety in emergency situations. There were two kinds of disclosure to authority figures. In the first type, YA informed their educators and employers that they had ESRD. Oftentimes, this process was spurred by an emergency situation or a scenario in which their illness posed an acute problem. In the second form, they actively discussed with them the accommodations they needed. YA described effective communication styles used to establish social support, such as maintaining direct contact with a manager, professor, or administrative personnel that dealt with matters of school or workplace disability. Additionally, they emphasized that these relationships were successful when employers and educators exercised understanding yet still held the individual accountable for their work.

Participants also discussed how they used these supports to ask for accommodations. Adjustments in both settings centered on schedule changes to allow for appointments and dialysis sessions. Requests for specific needs were related to participants’ knowledge of their disease, their physical limits, and the extent to which they felt their condition could impact their ability to attend school or work. The accommodation needs varied by symptomology, treatment modality, and type of employment or schooling. This adds to previous studies that identify support from supervisors as a means by which patients are able to engage in self-management behaviors, such as taking medications on site or asking for schedule adjustments [41]. Moreover, Mitchell et al. studied the transition to a dialysis regimen for adult renal patients and found that family and friends act as crucial sources of informational and emotional support for this population, which ultimately allows them to handle better the self-management task of hemodialysis adherence [42]. Our findings indicate that these networks extend further to include educators and employers who can also provide the support that facilitates self-management.

Overall, our study found that greater acceptance of disease coupled with knowledge of one’s limits and responsibilities can spur transition readiness behaviors, particularly those related to educational/vocational planning and interacting with relevant figures to enable these plans. The idea of progressing from an illness self-awareness phase into plan-making differs from models proposed by other scholars within the field of HCT. Reiss, Gibson, and Walker posit that “envisioning a future”, or the stage in which parents are tasked with imagining their child reaching adulthood, can encourage future planning and lead to the “age of responsibility” in which the child learns to care for their own disease-related needs [43]. The authors note that this process requires constant revisions as the disease itself changes the abilities and goals of a YA. While this model is not ineffective, our study suggests that when older patients are engaged in future planning, these steps should be reversed; it is more effective for the patient to recognize self-management needs before “envisioning a future.” The importance of prioritizing self-management can be seen across multiple research respondents in which a participant described an attempt to finish school or work was too stressful and ultimately harmful to their kidney function. While a goal or a sense of purpose is healthy for YA, our study revealed that career or educational pursuits could be harmful if carried out without self-care as the foundation of decision making.

Some participants described ways they avoided disclosure and support formation with an employer or educator due to fears of judgment and job loss. Findings were substantiated by a study conducted by Murray et al. that suggested that these fears could deter adults with ESRD and those with other chronic illnesses from asking authority figures for self-care adjustments [22]. Additional research reported that chronically ill young adults in school settings might hesitate to take medications in front of their peers, fearing that disclosure of their condition and seeming “different” could lead to teasing, bullying, and the loss of friends [26]. Kiernans and Maynooth also observed how renal patients hesitated to reveal disease information in social situations [38]. Within our study, participants did not report such severe social consequences but rather explained more subtle repercussions, including the sensationalization of their disease by others or having to answer questions they deemed “too personal,” particularly when trying to maintain professionality. Most notably, other participants affirmed the utility of disclosure for the sake of emergency situations. Consequently, a lack of disclosure could pose risks beyond social discomfort to patients who experience an emergency in a context where those around them are unaware of their condition.

There are several limitations to the current study. First, the sample was drawn from patients who had access to consistent care at a hospital in the Southeast United States, affecting the generalizability of results. Furthermore, the majority of the sample had never experienced transplantation and instead utilized some form of dialysis as their main treatment modality. There is a considerable difference between the disease management burden of dialysis and that of a transplant. Thus, it should be noted that the results of the current study were interpreted predominantly through the experience of dialysis. Moreover, given the current study’s small sample size, researchers were unable to investigate the relationship of treatment modality to onset-age or occupational status or level. Furthermore, only two participants responded to the invitation to review preliminary findings, themes, and direct quotations. While disappointing, this is not surprising given the complicated medical regimes that YA with ESRD must follow. Finally, as the participants were offered 40$ for their participation, the results may be biased towards the experiences of those who urgently needed monetary compensation.

The majority (82.6%) of eligible participants with whom contact was made agreed to complete the study. However, approximately half of individuals on the original list of eligible nephrology patients could not be reached. It is possible that patients had dropped out of care, moved, or listed a phone number that was out of service at the time of recruitment. In addition, findings reflect only the experiences of those willing to discuss their condition. Data are qualitative and self-reported. Findings do not include the perceptions of relevant figures such as parents, educators, employers, nor medical providers.

Further exploration is needed into the perspectives of authority figures in education and employment settings, particularly educators, school administrators, educational disability service providers, human resource departments, and employers. While the current study has provided a glimpse into exchanges with these figures from a patient’s point of view, additional insight is needed to understand how those in authoritative positions communicate with, perceive, and address the needs of renal patients. Future research should examine the structure and context of HCT behaviors and processes within particular models that allow for a comprehensive view of a patient. The International and Interdisciplinary HCTRC Healthcare Transition Model is an effective tool by which practitioners and caregivers can guide the multi-faceted development of YA with ESRD. This and other models must include education for patients regarding the academic and professional limitations they could face as a result of their condition. These limitations should also be discussed in conjunction with strategies to overcome such barriers, specifically those related to social support. YA must become aware of the utility of support as well as how it can be established. Additionally, ESRD patients in young adulthood should be prepared to discuss with educators and employers how to best care for themselves while also following their goals and aspirations. In turn, educators and employers must remain aware of patients’ needs and accommodate them where necessary.

## 5. Conclusions

Overall, the relationship between social support in education and employment settings and transition readiness appears to be symbiotic. Support in these areas facilitates transition in that it allows YA to carry out self-management behaviors while completing normal developmental tasks such as attending school and maintaining employment. At the same time, a successful transition process not only equips a patient with an understanding of their condition and disease-related needs but also builds their confidence in making choices independently and articulating these needs to important figures outside domestic or medical settings.

## Figures and Tables

**Table 1:** Participant Demographic Features.

Demographic Variable (n = 19)	M (Range, SD)
Age	24 (range 19–28, 2.6 years)
	n (%)
Gender	
Male	10 (52.6)
Ethnicity	
African American	9 (47.4)
Caucasian	6 (31.6)
Latino	3 (15.8)
American Indian	1 (5.2)
Employed	6 (31.6)
Part-time	3 (15.8)
Full-time	3 (15.8)
Unemployed	13 (68)
Living Situation	
With Parents	8 (42.1)
With Partner	6 (31.6)
Independently	5 (26.3)
Education Level	
Some high school	6 (31.6)
Some college/vocational school	12 (63.2)
Undergraduate degree	1 (5.2)
Learning Disabled	1 (5.2)
Income	
$5000–$10,000	9 (47.4)
$10,001–$20,000	3 (15.8)
$20,001–$40,000	3 (15.8)
More than $40,001	2 (10.5)
Unknown	2 (10.5)

**Table 2:** Disease-related Characteristics.

Characteristic (n = 19)	n (%)
Pediatric onset of end stage renal disease (ESRD)	8 (42.1)
Adult onset of ESRD	11 (57.9)
Has ever used dialysis	15 (78.9)
Using dialysis at time of study	13(68.4)
Hemodialysis	10 (52.6)
Peritoneal Dialysis	3 (26.3)
Had ever been transplanted	5 (26.3)
Transplanted at time of study	3 (15.8)
	M (range, SD)
Mean onset of dialysis (age)	20.1 (range 12–26, SD 4.2 years)
Mean duration of dialysis (years)	3.4 (range 1–13, SD 3.1 years)
Mean years with ESRD	6.7 (range 1–24, SD 7.2 years)
Mean years with chronic kidney disease	8.4 (range 1–24, SD 7.6 years))

## Data Availability

Data available upon request.

## Supplementary Materials

The following are available online at https://www.mdpi.com/article/10.3390/ijerph18126596/s1, File S1: EMERGING ADULT DEMOGRAPHIC INTERVIEW.
