# Supplementary material for: “It’s What I Have, It’s Not Who I Am”: A Qualitative Study of Social Support in Education/Employment Settings and Transition Readiness of Young Adults with End-Stage Renal Disease
Source: Int J Environ Res Public Health. 2021 Jun 19;18(12):6596. doi: 10.3390/ijerph18126596 (PMC8296423; doi:10.3390/ijerph18126596)
Supplement: Supplementary file 1 [file ijerph-18-06596-s001.zip › ijerph-1221438-supplementary.pdf]

EMERGING ADULT DEMOGRAPHIC INTERVIEW

ID# ___________________
Date ___________________
Interviewer ______________
Location ________________

1. How far have you gone in school? (follow up with age if didn't complete school)
   - ☐ 1= Less than high school
   - ☐ 2= Some high school (did not graduate)
   - ☐ 3= Currently in high school
   - ☐ 4= GED
   - ☐ 5= High school graduate
   - ☐ 6= Some college (did not graduate)
   - ☐ 7= Vocational school
   - ☐ 8= Currently in college
   - ☐ 9= Undergraduate degree
   - ☐ 10= Graduate school degree

2. While you were in school did you have an Individualized Education Plan?
   - ☐ 1= Yes
   - ☐ 2= No

3. Are you currently employed?
   - ☐ 1= Employed
   - ☐ 2= Unemployed

   a. Type of employment:
   ____

   b. Full or part-time status:
   - ☐1= full-time
   - ☐2= part- time

4. What is your age? ___________________________________
5. How do you describe your race/ethnicity? ________________
6. When were you diagnosed with chronic kidney disease? ____ ESRD? _____
7. Are you currently using dialyses? If so, hemodialysis or peritoneal?
8. Have you ever received a transplant? If so, do you still have that transplant?

- In school: Section A
- Employed: Section B
- Neither: Section C

Section A

- What are you studying?
- Why do you like it?
- How is your participation in school right now affected by your illness?
- What are your future goals for your education?
- Where do you find support for these goals? (prompt for people or programs)
- What kinds of things get in the way of these goals? (probe for illness-related challenges)

Effects of ESRD

- What are your experiences with managing your illness at school? (probe for good experiences and challenges)

Disclosure

- Who at school knows about your condition?
- How if at all, do you communicate with your educators regarding your condition?
- Who at school is available for you to talk to about your health issues? What resources are available?

Accommodations/Adjustments (classify as type of social support)

- When you're at school, what kinds of things do you need to ask from educators related to your condition in order to help you out? (i.e. schedule changes, foods you can eat, taking time off etc.)?
- Are all of these in place for you?
- How do you request these? What kind of systems are set in place to do so?
- Do you feel comfortable doing so?

Future

- What advice would you give to others your age with ESRD regarding going to school?

Section B

- What job do you hold
- Why do you like it?
- How is your work right now affected by your illness?
- What are your future goals in terms of your job or later career?
- Where do you find support for these goals?
- What kinds of things get in the way of these goals? (probe for illness-related challenges)

Management of ESRD

- What are your experiences with managing your illness at work? (probe for good experiences and challenges)

Disclosure

- Who at work knows about your condition?
- How if at all, do you communicate with your employers regarding your condition?
- Who at work is available for you to talk to about your health issues? What resources are available?

Accommodations/Adjustments (classify as type of social support)

- When you're at school, what kinds of things do you need to ask from employers related to your condition in order to help you out? (i.e. schedule changes, foods you can eat, taking time off etc.)?
- Are all of these in place for you?
- How do you request these? What kind of systems are set in place to do so?
- Do you feel comfortable doing so? Why or why not?

Future

- What advice would you give to others your age with ESRD regarding working?

Section C

- If not, what activities are you currently involved in?
- What do you enjoy doing with your time?
- What are your future goals with these activities?
- Where do you find support for these goals?
- What kinds of things get in the way of these goals? (probe for illness-related challenges)?

Effects of ESRD

- What are your experiences with managing your illness at (setting) probe for good experiences and challenges)

Disclosure

- Who at (setting) knows about your condition?
- How if at all, do you communicate with others in (setting) regarding your condition?
- Who at (setting) is available for you to talk to about your health issues? What resources are available?

Accommodations/Adjustments (classify as type of social support)

- When you’re at (setting), what kinds of things do you need to ask from (people in setting) related to your condition in order to help you out? (i.e. schedule changes, foods you can eat, taking time off etc.)?
- Are all of these in place for you?
- How do you request these? What kind of systems are set in place to do so?
- Do you feel comfortable doing so?

Future

- What advice would you give to another young person who has your same condition?
